# A self-affirmation exercise does not improve intentions to vaccinate among parents with negative vaccine attitudes (and may decrease intentions to vaccinate)

**DOI:** 10.1371/journal.pone.0181368

**Published:** 2017-07-13

**Authors:** Rachael D. Reavis, Jacob B. Ebbs, Adaobi K. Onunkwo, L. Mariah Sage

**Affiliations:** Department of Psychology, Earlham College, Richmond, Indiana, United States of America; University of Waterloo, CANADA

## Abstract

Two studies investigated the effectiveness of a self-affirmation exercise on vaccine safety beliefs and intent to vaccinate future children. In Study 1, a sample of 585 parents with at least one child under the age of 18 in the home participated through Amazon’s MTurk. Participants were randomly assigned to one of four conditions in a 2 x 2 design. Participants read either correcting information refuting a link between the measles, mumps, and rubella (MMR) vaccine and autism or a control passage about bird feeding. Additionally, participants either completed a self-affirmation exercise where they reflected on their personal values or in a control condition in which they reflected on least-personally-important values that might be important to others. Participants exposed to the correcting information were less likely to believe that vaccines cause serious side effects, but no less likely to believe that the MMR vaccine causes autism. For parents with initially positive vaccine attitudes, there was no effect of condition on intent to vaccinate a future child. For parents with initially negative vaccine attitudes, self-affirmation was ineffective in the presence of correcting information and resulted in less intention to vaccinate in the absence of correcting information. This effect was partially replicated in Study 2 (*N* = 576), which provided no correcting information but otherwise followed the same procedure as Study 1.

## Introduction

The Center for Disease Control and Prevention (CDC) recommends administration of 13 different vaccines for children ages 0 to 18 [1,2]. Vaccines are largely responsible for the impressive decline in childhood illnesses [3], many of which are now rare in the United States (e.g., polio, measles). However, lower-than-ideal vaccination rates in the U.S. are a growing public health concern [4], and have been implicated in several measles and pertussis outbreaks [3]. Despite the scientific evidence that vaccines are safe for the large majority of children and prevent childhood disease and mortality [5,6], some parents remain resistant to vaccinating their children. Researchers have tested various messaging techniques to decrease negative vaccine attitudes and to increase vaccination rates, but have found it difficult to change parents’ negative attitudes (e.g., [7,8]). The present study attempted to apply a psychological intervention—a self-affirmation exercise—which has proven helpful in health messaging acceptance in other domains (e.g., smoking, healthy eating) [9,10] to vaccine safety messaging for parents.

Fifteen U.S. states are below the *Healthy People 2020* target vaccination rate of 90% and 36 states are below the World Health Organization (WHO) recommendation rates of 93% vaccination rates to prevent epidemics [11,12]. From 2001 to 2010, there were eight years with fewer than 100 cases of measles in the U.S. and two years with fewer than 150 cases [13]. In 2011 and 2013 there were approximately 200 cases each year, and in 2014, there were over 600 cases in 23 outbreaks. In all outbreaks, the majority of infected individuals had not been vaccinated [13]. The largest outbreak in 2014 originated in California at Disneyland [14], and there are schools in California where fewer than 50% of kindergartners are up-to-date on their vaccines [15].

The rise in parents choosing not to vaccinate their children may be partially due to parental fears that vaccines cause serious side effects, including autism [16]. Although fears about vaccines have been present since their introduction [17], the wave of current fears is due in part to a discredited study by Andrew Wakefield, which purported to show a link between the MMR vaccine and autism [18]. Despite that fact that this study was eventually withdrawn and determined to be fraudulent [19] and that the scientific consensus is that vaccines are *not* related to the development of autism (e.g., [20,21]), vaccine safety concerns have persisted [22].

Nyhan and colleagues [7] tested the effect of several messaging techniques on negative attitudes toward vaccines (beliefs that they cause autism or other severe side effects) and likelihood to vaccinate future children. One technique was to expose parents to scientific evidence, similar to what is provided by the CDC, which refutes a link between the MMR vaccine and autism. Interestingly, although exposure to such information decreased agreement with statements that the MMR vaccine is unsafe, exposure also decreased intention to vaccinate future children, particularly among those with already negative attitudes toward vaccines. Thus, although the researchers found evidence of surface acceptance of the health messaging (in that beliefs about vaccine dangers decreased), the health message had a negative effect on intent to vaccinate. Parents with negative attitudes were paradoxically *less* likely to say they would vaccinate a future child than if they had been exposed to no messaging at all.

One possibility for this paradoxical effect is that the correction of misinformation may have threatened participants’ sense of self. We are generally motivated to maintain a positive sense of self [23], and for parents with negative attitudes toward vaccines, being exposed to conflicting scientific information could be perceived as threatening. In other areas of health messaging, participants who read health messages that are particularly relevant for their lives (e.g., obese people who are unhealthy eaters reading about the importance of healthy eating) may react defensively as if the health message is a personal attack on their self-worth [24]. For example, an obese person who is an unhealthy eater may interpret health messages about healthy eating as implying that they lack self-control or are ignorant, which would threaten their self-worth. Thus, they are more likely to dismiss the message.

When people affirm their sense of self, they are less sensitive to threats to their self-worth and may be more willing to accept health messages [24]. One method to help participants affirm and strengthen their sense of self is to have individuals reflect on their personal values and why they view them as important [25]. Individuals who self-affirm tend to see potentially threatening health messages as less threatening [24]. Self-affirmation may help individuals maintain a positive self-image and seems to improve the acceptance of health messages in the domains of fruit and vegetable consumption and smoking [26,27].

The effects of self-affirmation on health message acceptance seem to be largest for relevant high-risk groups. For example, high-risk participants who completed a self-affirmation exercise showed the most willingness to take a Type-2 diabetes risk test following a health message, compared to low-risk participants or high-risk participants who did not self-affirm [28]. Similar results have been seen with consumption of high-mercury seafood and caffeine consumption [29,30].

Self-affirmation may be an effective tool at increasing acceptance of vaccine safety messaging, particularly for those who have more negative vaccine attitudes (e.g., those for whom the messages are intended and are the most relevant). Based on research in other health domains, we would expect self-affirmed participants—particularly those with initially negative vaccine attitudes—to be more willing to accept the safety messaging, and perhaps be more willing to vaccinate future children.

However, there are important differences between vaccine safety messaging and other types of health messages. The first is that in the case of vaccines, parents are making health decisions not for themselves, but for their children. The effect of self-affirmation for health decisions made for *others* rather than the self has not been studied. Another difference is that parents with negative vaccine attitudes may have deeply held beliefs that vaccines are unsafe, whereas it is unlikely that individuals believe that cigarettes are good for you (as opposed to believe cigarettes are not as bad as health messaging claims) or that eating vegetables is an unhealthy lifestyle. It may be that self-affirmation will actually *strengthen* these negative attitudes and make participants less likely to accept vaccine safety messages. Given these differences, we do not make directional hypotheses testing the effect of self-affirmation on acceptance of vaccine health messaging, particularly for those with initially negative vaccine attitudes. In the absence of self-affirmation, we predict results similar to those of Nyhan et al. [7]. We expect that participants exposed to scientific information refuting the connection between the MMR vaccine and autism will be less likely to agree with statements that suggest vaccines are unsafe, but that those with initially negative vaccine attitudes will be less likely to say they would vaccinate a future child if exposed to correcting information versus a control condition. Again, for participants with negative vaccine attitudes, self-affirmation may strengthen or reverse these results.

## Study 1

### Method

The Earlham College IRB approved this research (IRB Approval Number: 1314-e027). All participants provided written (digital) consent.

#### Participants

Participants were 585 adults (47% male) with at least one child under the age of 18 living in the household. The median number of children in the home was one child (*M* = 1.65, *SD* = 0.88, range: 1–5 children). All participants were recruited through MTurk and were compensated $0.30, which was an effective hourly rate of $2.65. The consent form indicated that participation required having one child under the age of 18 living in the home. Originally, 680 participants opened the survey, but 15 did not advance past the consent form, and 37 did not advance past the first question where they indicated the number of children (28 of those indicated that they had no children at home). Six participants did not complete past the demographics. Fifteen participants indicated their initial attitudes and then stopped participating. Three participants completed the first experimental manipulation (self-affirmation), but did not advance to the second (correction information vs. control passage.) An additional 11 participants failed to agree with the statement, “I was honest in answering all questions (including about whether I have a child in the home),” with five disagreeing and six skipping the question. Six participants answered an attention check question incorrectly, and two participants did not follow instructions and failed to write about their values when prompted.

The sample was predominantly Caucasian (70%). Educational background was varied, ranging from participants who had not graduated from high school to participants with doctoral and professional degrees (47% had a 4-year college degree or above). The average age of parents was 32.76 years (*SD* = 7.9 years; range: 18–61 years). The majority of participants were married or cohabitating (76%). The majority of participants were from suburban areas (57%), followed by urban (25%), and rural (18%). Participants were from 48 U.S. states plus the District of Columbia. There were no participants from Montana or Vermont. There was a wide range of income, with 4% reporting annual incomes under $10,000 and 2% reporting incomes over $150,000, with a median income bracket of $40,000 to $49,999. Table 1 shows demographic comparisons between the sample of the current study and Nyhan and colleagues’ sample [7].

**Table 1:** Comparison of demographics between Nyhan et al. [7], Study 1, and Study 2.

	Nyhan et al.	Study 1	Study 2		Nyhan et al.	Study 1	Study 2
**Sex**				**Household Income**			
Female	56%	53%	60%	Less than $30,000	30%	26%	47%
Male	44%	47%	40%	$30,000-$49,999		28%	39%
				$30,000-$59,000	24%		
**Race/Ethnicity**				$50,000-$99,999		36%	14%
White	62%	70%	75%	$60,000-$99,999	25%		
Black	12%	12%	9%	$100,000+	20%	10%	0%
Hispanic	19%	7%	4%				
Other	4%	7%	6%	**Number of Children in Household**			
Multiracial	3%	4%	6%	1	39%	55%	56%
				2	39%	32%	32%
**Education**				3	15%	9%	8%
Not high school graduate	13%	1%	1%	4	5%	3%	3%
High school graduate	27%	9%	8%	5+	2%	1%	1%
Some college	29%	43%	43%				
College graduate	31%	47%	48%				
**Age**							
Less than 30	20%	40%	31%				
30–40	39%	42%	49%				
41 and older	41%	18%	20%				

#### Procedure

Participants completed the survey on Qualtrics, using a link on MTurk, in June 2014. A consent form indicated that the study was on memory and health decisions for children. Participation was restricted to parents/guardians of at least one child in the home under the age of 18. Following a demographic form, participants completed a measure of their vaccine attitudes [31], plus two questions about political affiliation. Statements were presented randomly. Participants were then randomly assigned to a self-affirmation or control condition using a standard values affirmation procedure (e.g., [25]). Participants were presented (in random order) with 11 qualities they might value (e.g., religious values, humor) and asked to either select the value that was most important (self-affirmation) or least important (control). They then either wrote about why they felt the value was important to them (self-affirmation) or to someone else (control). Finally, they answered four questions on a 6-point Likert scale (Strongly Disagree to Strongly Agree) about the importance of the value to themselves (self-affirmation) or to other people (control).

Following the values procedure, participants were randomly assigned to one of two reading passages, both of which were taken from Nyhan et al. [7]. They were told that they would answer questions about the passage later on, although they did not actually do this. The instructions were to increase the likelihood of close reading. In the Control condition, participants read about the costs and benefits of feeding birds. In the Autism Correction condition, participants read information from the CDC refuting the link between autism and the MMR vaccine. There were four total conditions: Autism Correction/Self-Affirmation (*N* = 156), Autism Correction/Values Control (*N* = 139), Control Passage/Self-Affirmation (*N* = 138), and Control Passage/Values Control (*N* = 152).

Participants were presented (in random order) three outcome questions measuring beliefs about the MMR vaccine and autism (also from Nyhan et al. [7]) and an attention check question, which asked participants to select “Neutral” on a “Strongly Disagree” to “Strongly Agree” scale to demonstrate that they had been paying attention. After entering their MTurk ID, participants were asked to agree or disagree with a statement about whether they had answered honestly and were told that their answer would not affect their payment (and it did not). Participants were then debriefed. The procedure with individual questions is in the appendix.

#### Measures

*Pre-intervention vaccine attitudes* were assessed using 10 questions from Freed et al. [31], answered on a 5-point Likert scale from Strongly Disagree to Strongly Agree, such as “Getting vaccines is a good way to protect my child(ren) from disease,” and “Some vaccines cause autism in healthy children” (reverse scored). The scale was reliable, α = .89. Participants who discontinued after indicating their attitudes had more negative attitudes (*M* = 3.95, *SD* = 0.85) than participants who completed the study (*M* = 4.31, *SD* = 0.79; *t*(620) = -2.74, *p* = .006), suggesting that the eventual sample was biased toward those with more positive views of vaccines.

To examine the effect of condition based on initial vaccine attitudes, Nyhan and colleagues used a tercile split. Participants whose vaccine attitudes scores were less than 3.57 were classified as having “least favorable attitudes,” (and referred to as “negative” in the conclusion) even though their score is closer on the scale to “Agree” than to “Neither Agree or Disagree.” (Those with scores greater than or equal to 4.125 were classified as having “most favorable” attitudes.) These cut-offs resulted in those with “least favorable” attitudes having a *group* mean of 3.0 (“Neither Agree Nor Disagree”). We felt it was important to examine negative attitudes and to use an objective, rather than relative, cut-off for determining those negative attitudes. Thus, we divided participants based on their position on the scale, not based on their position *relative* to other participants. This approach resulted in participants being classified into a *positive vaccine attitude* group (individual mean scale score at or above 4 or “Agree,” *N* = 399, group *M* = 4.74, *SD* = 0.44), a *negative vaccine attitude* group (individual mean scale score below 3 “Neither Agree Nor Disagree,” *N* = 38, group *M* = 2.51, *SD* = 0.43), or being removed from analyses (*N* = 148). However, we have included analyses using both approaches.

Following Nyhan et al. [7], *autism belief* was measured with a single item, answered on a 5-point Likert-scale (Strongly Disagree to Strongly Agree): “Some vaccines cause autism in healthy children.” *Side effects belief* was measured with a single item, answered on a 6-point Likert-scale (Very Unlikely to Very Likely): “How likely do you think it is that a child will suffer from serious side effects from the measles, mumps, and rubella (MMR) vaccine?” *Intent to vaccinate* was measured with a single item on the same 6-point scale: “If you had another child, how likely is it that you would give that child the measles, mumps, and rubella vaccine, which is known as the MMR vaccine?”

### Results

Table 2 displays the descriptive statistics for Study 1 variables. Using a chi-square, we tested whether participants who were excluded after the experimental manipulations were more likely to have been assigned to one condition over another. We saw no support for this, χ^2^ = 1.96, *p* = .58. We examined the effect of conditions on each of three outcomes: *autism belief*, *side effects belief*, and *intent to vaccinate*. In each case, we first ran a 2 (passage condition: autism correction or control) x 2 (affirmation condition: self-affirmation or control) ANOVA with the full sample. Then we conducted a 2x2x2 ANOVA with the vaccine attitude group as a third variable, which involved a subset of the sample and did not include participants with moderate vaccine attitudes.

**Table 2:** Means & Standard deviations of Study 1 variables.

	Total	Control Passage		Autism Correction	
		Self-Affirm	Control	Self-Affirm	Control
Initial Vaccine Attitudes	4.31 (0.79)	4.35 (0.76)	4.34 (0.78)	4.27 (0.81)	4.30 (0.80)
Autism Belief	2.22 (1.22)	2.20 (1.18)	2.23 (1.23)	2.21 (1.25)	2.23 (1.22)
Side Effects Belief	2.26 (1.22)	2.36 (1.25)	2.39 (1.33)	2.21 (1.15)	2.09 (1.13)
Intent to Vaccinate	5.06 (1.39)	5.09 (1.36)	5.05 (1.48)	4.98 (1.38)	5.12 (1.34)

*Note*. *Initial vaccine attitudes* [31] were measured based on 10 questions answered on a 5-point scale (Strongly Disagree to Strongly Agree), such that higher numbers indicate more positive vaccine attitudes. *Autism belief* was measured using a 5-point scale (Strongly Disagree to Strongly Agree) in response to the question: “Some vaccines cause autism in healthy children.” *Side effects belief* and *Intent to vaccinate* were measured on a 6-point scale (Very Unlikely to Very Likely) in response to the questions: “How likely do you think it is that a child will suffer from serious side effects from the measles, mumps, and rubella (MMR) vaccine?” and “If you had another child, how likely is it that you would give that child the measles, mumps, and rubella vaccine, which is known as the MMR vaccine?” respectively. These questions were drawn from Nyhan et al. [7]. Reading passages were drawn from Nyhan et al. [7]. The values affirmation procedure was drawn from Sherman et al. [25].

#### Autism belief

We found no main effect of passage condition on beliefs that the MMR vaccine causes autism, *F*(1,579) = 0.06, *p* > .250, which was in contrast to Nyhan et al. [7]. Likewise, there was no main effect of affirmation condition on *autism belief*, nor was there an interaction with passage condition, *F*s(1, 579) < 0.10, *p*s > .250. Using the subsample based on vaccine attitudes, we found no significant three-way interaction between passage condition, affirmation condition, and initial vaccine attitudes on *autism belief*, *F*(1, 427) = 0.18, *p* > .250. We also tested the three-way interaction using Nyhan’s cut-offs for relative vaccine attitudes. This interaction was not statistically significant, *F*(1, 461) = 2.32, *p* = .128.

#### Side effects belief

Unlike Nyhan et al. [7], we found a small main effect of passage condition on *side effects belief*, *F*(1, 580) = 4.95, *p* = .026, η^**2**^_**p**_ = 0.01, such that participants who read the autism correction passage agreed less with the statement that the MMR vaccine is likely to cause serious side effects (*M* = 2.15, *SD* = 1.14, 95% CI: 2.01–2.29) than did participants who read the control passage (*M* = 2.38, *SD* = 1.29, 95% CI: 2.23–2.52). There was no main effect of affirmation condition and there was no interaction with passage condition, *F*s(1, 580) < 1.0, *p*s > .250. Using the subsample based on vaccine attitudes, we found no significant three-way interaction between passage condition, affirmation condition, and initial vaccine attitudes on *side effects belief*, *F*(1, 429) = 0.07, *p* > .250. Similarly, there was no statistically significant three-way interaction using Nyhan’s cut-offs for relative vaccine attitudes, *F*(1, 462) = .14, *p* > .250.

#### Intent to vaccinate

**Fig 1:**
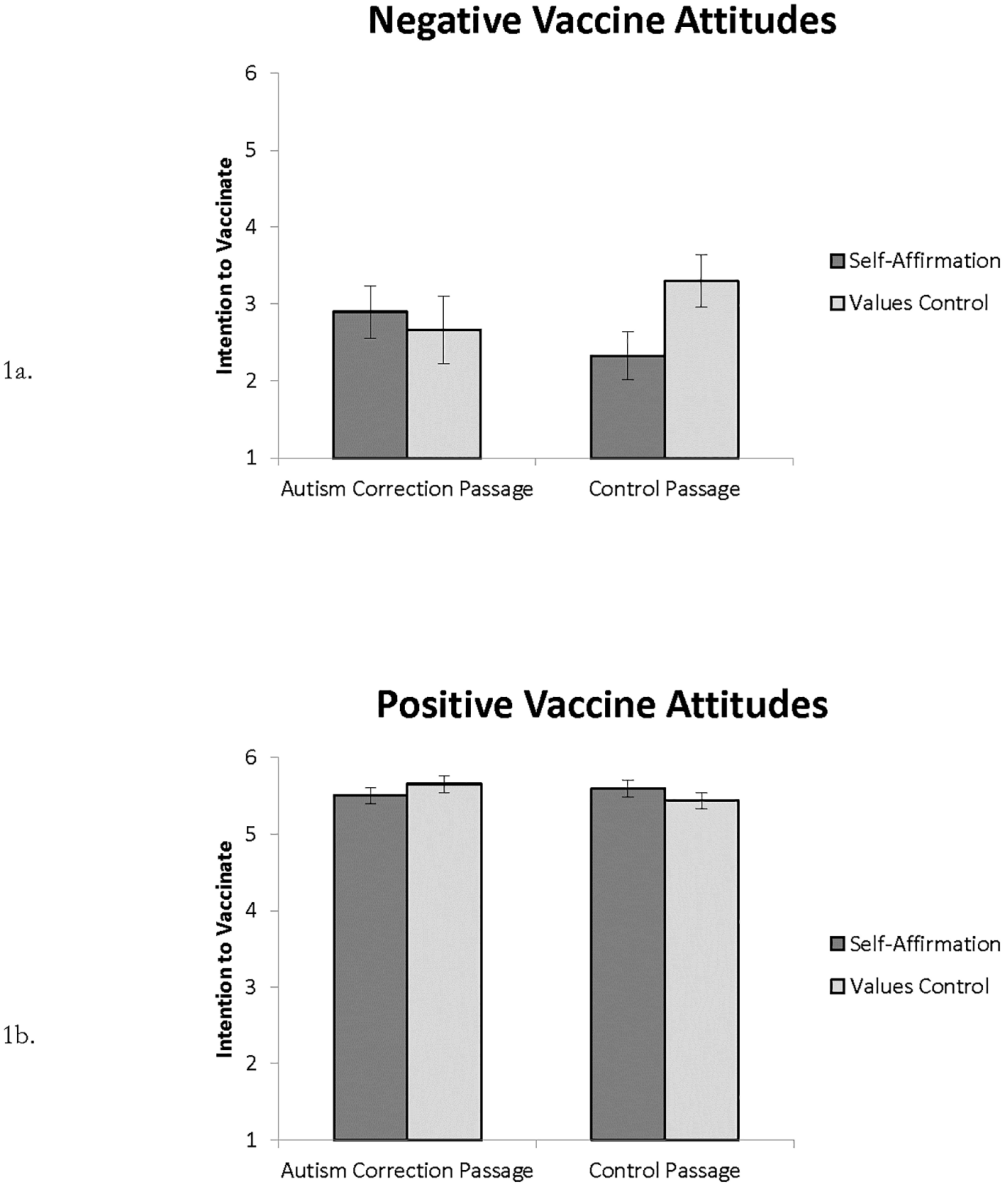
Depiction of three-way interaction between passage condition, affirmation condition, and initial vaccine attitudes. Vaccine attitudes [31] were measured on a 5-point scale (Strongly Disagree to Strongly Agree). (1a) Participants with mean attitude score lower than 3. (1b) Participants with a mean attitude score of 4 or greater. *Intent to vaccinate* was answered on a 6-point scale (Very Unlikely to Very Likely). Reading passages were drawn from Nyhan et al. [7]. The values affirmation procedure was drawn from Sherman et al. [25]. Error bars represent ±1 *SE*.

Whereas Nyhan et al. [7] found that reading the autism correction passage *decreased* the intent to vaccinate a future child, we found no significant main effect of passage condition on *intent to vaccinate*, *F*(1, 578) = 0.03, *p* > .250. There was no main effect of affirmation condition and there was no interaction with passage condition, *F*s(1, 580) < 1.0, *p*s > .250. Using the subsample based on vaccine attitudes, we found a significant three-way interaction between passage condition, affirmation condition, and initial vaccine attitudes on *intent to vaccinate*, *F*(1, 426) = 4.02, *p* = .046, η^**2**^_**p**_ = 0.01 (see Fig 1). For participants with initial positive vaccine attitudes, there was little difference between conditions. For participants with initial negative vaccine attitudes, values affirmation *decreased* intentions to vaccinate in the absence of correcting information (i.e., in the control passage condition) and had no effect in the presence of it (i.e., in the autism correction condition).

The analysis using Nyhan’s cut-offs for relative vaccine attitudes was not statistically significant (*F*(1, 460) = 1.31, *p* > .250), but the pattern was similar.

## Study 2

We conducted a second study to attempt to replicate our result that in the absence of correcting information, self-affirmation may increase parental resistance to vaccines among those with initially negative attitudes. Because we were interested in parents’ responses in the absence of correcting information, we dropped the passage condition. Thus, all participants either self-affirmed or did not and no participants in Study 2 saw correcting information about the safety of vaccines.

### Method

#### Participants

Participants were 576 adults (40% male) with at least one child under the age of 18 living in the household. The median number of children living in the household was one child (*M* = 1.62, *SD* = 0.88, range: 1–7 children). All participants were recruited through MTurk and were compensated $0.30, which was an effective hourly rate of $2.65. The consent form indicated that participation required having one child under the age of 18 living in the home. Originally, 751 participants opened the survey, but 37 did not advance past the consent form. Eighty-five participants indicated they had no children, did not give their children’s ages, or did not advance past the question asking about children. Five participants did not complete past the demographics. Nineteen participants indicated their initial attitudes and stopped participating. An additional 10 participants failed to agree with the statement, “I was honest in answering all questions (including about whether I have children in the home).” Five participants answered an attention check question incorrectly, and two participants did not follow instructions and failed to describe values when prompted. Finally, we compared MTurk identification numbers between Study 1 and Study 2. Twelve participants were excluded from analyses of Study 2 because they had previously participated in Study 1.

Although less pronounced than in Study 1, there was evidence that the eventual sample was biased toward those with more positive views of vaccines. Excluded participants had more negative attitudes (*M* = 4.18, *SD* = 0.76) than included participants (*M* = 4.39, *SD* = 0.85; *t*(624) = -1.71, *p* = .087).

The sample was predominantly Caucasian (75%). Educational background was varied, ranging from participants without a high school diploma to participants with a doctoral and professional degrees (48% had a 4-year college degree or above.) The average age of parents was 34.98 years old (*SD* = 9.09 years; range: 18–64 years). The majority of participants were married or cohabitating (76%). The majority of participants were from suburban areas (59%), followed by urban (21%) and rural (20%) areas. Participants were from 47 U.S. States. There were no participants from New Mexico, Vermont, or Wyoming. There was a wide range of income, with 11% reporting annual incomes under $10,000 and 3% reporting incomes over $75,000, with a median income bracket of $30,000 to $39,000. Table 1 shows the demographic comparisons between samples of Nyhan and colleagues [7], Study 1, and Study 2.

#### Procedure

The procedure for Study 2 was identical to Study 1, except that there was no reading passage. There were two experimental groups: Self-Affirmation and Control. Participation occurred in August 2015.

### Results

Table 3 displays the descriptive statistics for Study 2 variables. We examined the effect of condition on each of three outcomes: *autism belief*, *side effects belief*, and *intent to vaccinate*. In each case, we first ran a t-test comparing the Self-Affirmation and Control groups. Then we conducted a 2 (affirmation condition: self-affirmation or control) x 2 (initial vaccine attitudes: positive or negative) ANOVA, which involved a subset of the sample and did not include participants with moderate vaccine attitudes.

**Table 3:** Means & Standard deviations of Study 2 variables.

	Total	Control	Self-Affirm
Initial Vaccine Attitudes	4.39 (0.85)	4.38 (0.80)	4.40 (0.90)
Autism Belief	2.16 (1.19)	2.19 (1.14)	2.13 (1.23)
Side Effects Belief	2.22 (1.29)	2.27 (1.29)	2.17 (1.29)
Intent to Vaccinate	5.16 (1.43)	5.19 (1.36)	5.11 (1.50)

*Note*. *Initial vaccine attitudes* [31] were measured based on 10 questions answered on a 5-point scale (Strongly Disagree to Strongly Agree), such that higher numbers indicate more positive vaccine attitudes. *Autism belief* was measured using a 5-point scale (Strongly Disagree to Strongly Agree) in response to the question: “Some vaccines cause autism in healthy children.” *Side effects belief* and *Intent to vaccinate* were measured on a 6-point scale (Very Unlikely to Very Likely) in response to the questions: “How likely do you think it is that a child will suffer from serious side effects from the measles, mumps, and rubella (MMR) vaccine?” and “If you had another child, how likely is it that you would give that child the measles, mumps, and rubella vaccine, which is known as the MMR vaccine?” respectively. These questions were drawn from Nyhan et al. [7]. The values affirmation procedure was drawn from Sherman et al. [25].

#### Autism belief

As with Study 1, there was no main effect of affirmation condition on *autism belief*, *t*(573) = 0.64, *p* > .250. There was no interaction between affirmation condition and initial vaccine attitudes, *F*(1, 455) = 0.01, *p* > .250. Likewise, there was no interaction when using Nyhan’s cut-offs for relative vaccine attitudes, *F*(1, 485) = 0.11, *p* > .250.

#### Side effects belief

As with Study 1, there was no main effect of affirmation condition on *side effects belief*, *t*(574) = 0.93, *p* > .250. Unlike Study 1, there was an interaction between affirmation condition and initial vaccine attitudes, *F*(1, 456) = 5.08, *p* = .025, η^**2**^_**p**_ = .01. For participants with initial positive vaccine attitudes, there was little difference between conditions. For participants with initial negative vaccine attitudes, values affirmation *increased* belief that vaccines cause side effects. The same pattern was observed in Study 1, but for intent to vaccinate (which was decreased for those with negative vaccine attitudes who self-affirmed.) The analysis using Nyhan’s relative vaccine attitude cut-offs was not statistically significant, *F*(1, 486) = 0.12, *p* > .250.

#### Intent to vaccinate

As with Study 1, there was no main effect of affirmation condition on *intent to vaccinate*, *t*(572) = 0.68, *p* > .250. Unlike Study 1, there was no interaction between condition and initial vaccine attitudes, *F*(1, 455) < .01, *p* > .250. In contrast, there was a small and marginally statistically significant result when using Nyhan’s cut-offs for relative vaccine attitudes, *F*(1, 484) = 2.62, *p* = .106, η^**2**^_**p**_ = .005. Condition had no effect on those with initial relative positive vaccine attitudes (*M* = 5.66 for both conditions), but for those with initial relative negative vaccine attitudes, their intent to vaccinate was lower in the self-affirmation condition (*M* = 3.06, *SE* = 0.16) compared to those in the control condition (*M* = 3.78, *SE* = 0.17).

## Discussion

We attempted to extend and explore the results of Nyhan et al. [7] regarding the effect of scientific information about vaccine safety on parents’ beliefs about vaccines and their intent to vaccinate future children. Nyhan and colleagues’ study demonstrated paradoxical results such that exposure to correcting information decreased beliefs about negative effects of the MMR vaccine (specifically that it causes autism), but also decreased intentions to vaccinate future children among those with initially negative attitudes toward vaccines. We tested a psychological intervention—self-affirmation—which has increased acceptance of health messages in other domains. The results of our studies partially replicated Nyhan et al., in that exposure to correcting information decreased agreement about the negative effects of vaccines (in our case, that vaccines are likely to cause severe side effects). Our results provide no evidence for the hypothesis that self-affirmation would be an effective strategy. Given the relatively few participants with negative vaccine attitudes, the results provide *weak* evidence that self-affirmation may actually be detrimental for parents with initially negative vaccine attitudes.

### Correcting information

Study 1 used two of the same reading passages that Nyhan and colleagues [7] presented to their participants. In addition to a control passage about bird feeding, we presented participants with scientific information refuting the connection between the MMR vaccine and autism, similar to what is provided by the CDC. Both studies found evidence that exposure to the correcting information was more effective at reducing agreement with statements suggesting vaccines are unsafe, although there were differences. Nyhan and colleagues found that the correcting information decreased agreement that the MMR vaccine causes autism but not with the statement that vaccines are likely to cause severe side effects. We found that correcting information decreased beliefs that the vaccine causes severe side effects, but not beliefs that the vaccine causes autism. The effect in our study was very small. Neither study showed that results differed based on initial vaccine attitude.

The reason that the pattern of results for these two questions differed between studies is not entirely clear. In both studies, these measures are correlated (Study 1: *r*(582) = .541; Study 2: *r*(575) = .562, *p*s < .001) and seem to be assessing a similar construct of negative vaccine outcomes. To allow comparisons across studies, we chose to separate these items. In future studies, researchers may want to create a scale with several items, increasing reliability in a single measure. It is also possible that demographic differences contributed. Our study sample was younger, whiter, more educated (although there was a smaller percentage of families in the highest income brackets), and more likely to have only one child compared to Nyhan and colleagues’ sample. Hispanic parents (19% of Nyhan’s sample; 7% of our Study 1 sample) are more likely than white non-Hispanic parents to believe that vaccines cause autism [31]. They are also more likely to believe that vaccines cause serious side effects, although the ethnic difference for this question is smaller. Furthermore, first born children are more likely to be fully immunized compared to their younger siblings [32], and the views of parents of singletons may differ and be differentially susceptible to vaccine messages. Although the pattern differed based on specific question, the results of both studies suggest that exposure to correcting information decreases beliefs that vaccines are unsafe.

### Self-affirmation

Nyhan and colleagues [7] found that for parents with initially negative attitudes toward vaccines, reading correcting information actually decreased intent to vaccinate a future child. In Study 1, we did not find a two-way interaction between passage type (correcting information or control) and initial vaccine attitudes. However, we did find a three-way interaction between passage type, initial vaccine attitudes, and self-affirmation condition (self-affirmation or control) in Study 1, and an interaction between initial vaccine attitudes and self-affirmation condition in Study 2 (which provided no correcting information).

In the presence of correcting information (Study 1), completing a self-affirmation exercise was ineffective for parents with initially negative vaccine attitudes. That is, for parents with negative vaccine attitudes who read correcting scientific information, their intent to vaccinate a future child did not differ if they completed the values affirmation exercise for themselves (self-affirmation) or not. In the *absence* of correcting information (Studies 1 & 2), completing a self-affirmation exercise resulted in *lower* intention to vaccinate a future child among parents with initially negative vaccine attitudes. As with the previous result, the effect of this interaction was very small.

It is important to note that we used two definitions of initially negative attitudes. We used both an absolute measure, in which those classified as having negative attitudes had mean scores on the “disagree” side of the scale. We also used a relative measure, based on Nyhan’s cut-off scores using a tercile split to allow for comparisons between studies. The different analyses resulted in different specific results (a significant interaction with vaccine attitudes in Study 1 using absolute, but not relative, attitudes; a significant interaction in Study 2 using relative, but not absolute, attitudes), but taken as a whole point to the same general conclusion: there is weak and somewhat inconsistent evidence that self-affirmation results in more negative vaccine attitudes for those without initially positive attitudes.

Although self-affirmation exercises seem to be effective tools in other areas of health messaging (e.g., smoking, healthy eating) [9,10], important differences led us to propose competing hypotheses. First, in other health domains, participants are making health decisions for themselves, rather than for their children. In the case of childhood vaccines, parents are making decisions for their children. Second, although high-risk individuals (e.g., smokers, those with poor diet) may downplay the warnings in health messages (e.g., likelihood of disease), they are unlikely to strongly believe that smoking is *good* for them or that healthy eating is *bad* for them. In contrast, parents with negative vaccine attitudes may not only downplay the benefits of vaccines for their children, but also sincerely believe that vaccines are unsafe and dangerous for their children. Thus, although it was possible that self-affirmation would affect vaccine safety message acceptance in the same ways that it affects other health messages, the results are more suggestive of the alternative hypothesis. Specifically, engaging in self-affirmation might have made parents more secure in their negative attitudes, particularly in the absence of any correcting information.

These results have implications both for vaccine attitudes and messaging, as well as for research applying self-affirmation to health issues in general. The current studies add to the body of literature suggesting that vaccine attitudes are difficult to change (e.g., [8]) and combined with Nyhan et al. [7], suggest that interventions with parents who have particularly negative vaccine attitudes may be more detrimental than doing nothing. Of course, in the current studies, there were few parents with negative vaccine attitudes using an absolute cut-off (*N* = 38 divided between four groups in Study 1; *N* = 41 divided between two groups in Study 2), and we should reach conclusions cautiously.

Researchers, health providers, and public health officials should continue to develop and test potential interventions. Although the self-affirmation procedure appears detrimental, there are value-focused approaches that may be effective. Self-affirmation, in this study and in previous studies, has generally involved reflecting on potentially unrelated values with the goal of strengthening the sense of self. Researchers in Australia (e.g., [33]) developed a public health decision-making tool for parents that requires parents to rate how important various vaccine-specific values are, such as “my child will be better protected from the potentially serious complications of these diseases” and “if my child experiences a severe complication, I may feel guilty or responsible because I had them vaccinated.” Using pre/post tests, researchers found that using the decision-making aid increased parents agreement that they were “leaning toward” vaccination [34]. Understanding how general values-affirmation versus the use of problem-specific (in this case, vaccine-specific) values affect decision making will be an important avenue of future research.

Given the importance of high vaccination rates for public safety and public health, researchers and public health officials will have to continue to explore methods to increase parent confidence in vaccines and increase vaccination rates. Recent outbreaks may naturally increase vaccination rates. When choosing whether to vaccinate their children, many parents engage in a risk-benefit assessment. Those who choose not to vaccinate may see the risk of getting childhood diseases as very low and thus emphasize the risks of vaccines [35]. The recent measles outbreaks have garnered nation-wide media attention. This attention may increase parents’ perceptions of the likelihood that their children could be exposed to measles, potentially altering the risk-benefit analysis of parents who previously refused vaccines for their children. In turn, more parents may choose to have their children vaccinated.

The current study also suggests future avenues of research for applying self-affirmation to health behaviors and decision-making. No studies to our knowledge have explored the use of self-affirmation in health decisions about others (children, incapacitated adults). Nor have researchers investigated self-affirmation as applied to health decisions where individuals may hold beliefs opposite to public health officials. That is, the effect of self-affirmation may be quite different for a person who simply believes that health messages are exaggerated (e.g., believing that smoking leads to cancer less often than warnings imply) versus believing that health messages are wrong (e.g., believing that vaccines are dangerous when health messages say the benefits far outweigh potential costs). Researchers might investigate the effect of self-affirmation on other deeply held beliefs that may conflict with some health or scientific messages, such as individuals who strongly believe that genetically modified organisms (GMOs) are dangerous for their health although the U.S. Food and Drug Administration maintains that they are safe [36]. Another example might be the subset of skiers who state they do not wear helmets because the helmets interfere with vision (and thus safety), although this concern is not borne out by the data [37].

The interpretation of the results is limited in some ways. First, although we had a relatively large sample size in both studies, only a small percentage (Study 1: 6%, *N* = 38; Study 2: 7%, *N* = 41) was classified as having negative vaccine attitudes, using the absolute cut-off approach. Although significant interactions were identified in both studies, it is important to note that estimates are less stable with smaller sample sizes [38], and further replication would be important. Future researchers may want to recruit participants in a way that will allow over-sampling of those with negative vaccine attitudes, allowing for a more adequately powered examination of those with truly negative vaccine attitudes (and not just relatively less favorable attitudes). For example, recruiting parents from pre-schools with low vaccination rates and on message boards or groups whose members are opposed to vaccines.

Second, we did not include any measure of participants’ suspicions or distrust of the study’s intent. Advocates on both sides of the vaccination argument accuse the other side of being propaganda [39], and participants with negative vaccine attitudes may be particularly suspicious of the researchers’ intent. As an anonymous reviewer suggested, this could result in a strengthening of previously held beliefs. Future researchers should directly assess this possibility.

Third, the measure of intent to vaccinate is an imperfect one. People are often not good at predicting their future behavior [40], and a better measure would be parents’ actual vaccination behaviors. Given that this intervention was ineffective at best and harmful at worst, it would not be appropriate to implement it in pediatricians’ offices. However, as researchers progress and identify more effective interventions, an important test of them will be if parents actually increase their vaccination rates following interventions, which might be implemented during or prior to well-child visits.

Despite these limitations, the study offers a reasonable extension of the Nyhan et al. [7] study, supporting their results overall (that correcting information can decrease belief in misinformation but interventions can be detrimental for parents with negative vaccine attitudes), although the pattern of results for specific questions differed. As demonstrated before, parental vaccine attitudes are difficult to shift. The study also offered a reasonable test of competing hypotheses about the effect of self-affirmation for health messaging, and suggests intriguing new avenues of research that may expand our knowledge of the effectiveness of self-affirmation exercises and under what circumstances they do and do not work.

## Appendix

### MTurk HIT description

Participants accessed the survey link through the HIT (Human Intelligence Task). Anyone MTurk worker residing within the United States and having a 95% approval rating could have seen the HIT description: “Complete surveys testing memory & asking about health decisions for children—open only to parent/guardians with children under age 18 living in the home—takes about 5 minutes to complete.”

### Initial Information / Procedure

Participants read a consent form indicating that the study was on memory and health decisions for children. Participation was restricted to guardians/parents of at least one child under the age of 18 in the home. Following the consent form, participants answered demographic questions.

### Vaccine attitude questions (plus political identification)

(Freed, Clark, Butchart, Singer, & Davis, 2010)

Questions (presented in random order) answered on 5-point scale: Strongly Disagree, Disagree, Neither Agree or Disagree, Agree, Strongly Agree.

Items marked with an asterisk (*) are reverse-scored.

- Getting vaccines is a good way to protect my child(ren) from disease
- Generally, I do what my doctor recommends about vaccines for my child(ren)
- I am concerned about serious adverse effects of vaccines*
- Parents should have the right to refuse vaccines that are required for school for any reason*
- Some vaccines cause autism in healthy children*
- My child(ren) does(do) not need vaccines for diseases that are not common anymore*
- New vaccines are recommended only if they are as safe as older vaccines
- Vaccines are important to me, personally
- I have delayed a recommended vaccination for one or more of my children*
- I have refused a recommended vaccination for one or more of my children*
- I consider myself politically liberal
- I consider myself politically conservative

### Values affirmation procedure

(Sherman, Cohen, Nelson, Nussbaum, Bunyan, & Garcia, 2009)

The conditions (self-affirmation or control) were identical except for the information in brackets. The first item in the bracket was administered to the self-affirmation condition and the second item was administered to the control condition.

- People place a high value on different qualities. Please read each quality in the list below and think about it. Then select the one that is [**most important** / **least important**] to you. [There may be several that are important, but select the one that is the most important to you at the moment. / You may find them all important to you, but select the one that is the least important to you at the moment.]
  *The following options were presented in random order to both groups.
  - Athletic ability
  - Creativity
  - Independence
  - Living in the moment
  - Membership in a special group (such as your community, racial group, or church group, among others)
  - Music
  - Politics
  - Relationship with friends
  - Relationship with family
  - Religious values
  - Sense of humor
- Look at the value that you picked as [**most important to you** / **least important to you**]. [Describe in a few sentences why that quality is the most important to you. / Think about a time when this quality might be important to **someone else**. Describe in a few sentences why that quality might be important to **someone else**.] Focus on your thoughts and feelings, and don’t worry about spelling, grammar, or how well written it is.
- Select how strongly you agree or disagree with each statement regarding the quality/value you selected as [most / least] important to you.
  *The following questions were presented in random order on a 6-point scale: *Strongly Disagree*, *Disagree*, *Somewhat Disagree*, *Somewhat Agree*, *Agree*, *Strongly Agree*
  - This value has influenced [my life / some people].
  - In general, [I / some people] try to live up to this value.
  - This value is an important part of who [I am / some people are].
  - [I / some people] care about this value.

### Passage procedure [Study 1 only]

Participants in both groups read: “You will now read a short passage & be asked questions later in the survey. You may read one of several different types of passages.”

Both passages were taken from Nyhan et al., 2014.

### Autism correction passage

*Please examine the following information about measles*, *mumps*, *and rubella carefully*. *You will be asked questions afterward*.

All children should be vaccinated for measles, mumps, and rubella. The measles, mumps, and rubella vaccine (MMR) is safe and effective.

Because signs of autism may appear around the same time children receive the MMR vaccine, some parents may worry that the vaccine causes autism. Vaccine safety experts, including experts at the Centers for Disease Control (CDC) and the American Academy of Pediatrics, agree that MMR vaccine is not responsible for recent increases in the number of children with autism. A 2004 Institute of Medicine report concluded that there is no link between autism and MMR vaccine, and that there is no link between autism and vaccines that contain thimerosal as a preservative.

Many scientific studies have found no link between MMR vaccine and autism. These studies include:

- A September 2008 study published in Public Library of Science was conducted to determine whether results from an earlier study claiming to find measles virus RNA in the intestinal tissue of autistic children could be confirmed. The results could not be confirmed, and no link between MMR and autism was found.
- A 2006 study published in the Journal of Autism and Developmental Disorders of 351 children with autism and 31 typically-developing children did not find a link between MMR vaccination and autism.
- A 2002 study by CDC in the New England Journal of Medicine followed more than 500,000 children and found no association between MMR vaccination and autism.

### Control passage

*Please examine the following information about bird feeding carefully*. *You will be asked questions about it afterward*.

Q: What are the costs and benefits of bird feeding?

A: It is difficult to assess the costs and benefits of bird feeding because it is difficult to compare the health of birds without access to feeders to the health of birds with frequent access to feeders. Only one study was able to obtain some sound results. That study found that any benefits of feeding only appear to occur sporadically under extreme climactic conditions. No research has been able to demonstrate a cost. Aside from costs and benefits to birds, there is a cost and benefit to humanity. The costs are obvious—the expense of bird feeding supplies.

The benefits include learning more about birds and the joy of connecting with the natural world. Bird feeding provides a direct, intimate view of the natural world for more than 50 million Americans who feed the birds in their yards. It is most popular in winter, when birds seem to need the most help. Some people worry that birds will suffer unless they make great efforts to the feeder filled, but research indicates that most birds do not depend on feeders.

### Outcome measures

All participants were presented with three outcome questions plus an attention question. These were presented in random order.

- How much do you agree with this statement? “Some vaccines cause autism in healthy children”
  *Answered on a 5-point scale: *Strongly Disagree*, *Disagree*, *Neither Agree nor Disagree*, *Agree*, *Strongly Agree*
- How likely do you think it is that a child will suffer serious side effects from the measles, mumps, and rubella (MMR) vaccine?
- If you had another child, how likely is it that you would give that child the measles, mumps, and rubella vaccine, which is known as the MMR vaccine?
  *Both answered on a 6-point scale: *Very Unlikely*, *Unlikely*, *Somewhat Unlikely*, *Somewhat Likely*, *Likely*, *Very Likely*
- Please select “neutral” for this question to demonstrate that you have been paying attention.
  *Options included: *Strongly Disagree*, *Disagree*, *Neutral*, *Agree*, *Strongly Agree*

### Additional Questions/Procedures

Participants entered their MTurk ID and answered the question below, after which they received their MTurk code, and read a debriefing statement. [To maintain confidentiality, the MTurk ID is not included in the open data set.]

- Agree or disagree with the statement: “I was honest in answering all the questions (including about whether I have a child in the home.)” Your answer to this question **WILL NOT** affect your payment, but it is important for us to know for the integrity of our study.
  *Options were “Agree” and “Disagree”
